# Supplementary material for: Cloud BioLinux: pre-configured and on-demand bioinformatics computing for the genomics community
Source: BMC Bioinformatics. 2012 Mar 19;13:42. doi: 10.1186/1471-2105-13-42 (PMC3372431; doi:10.1186/1471-2105-13-42)
Supplement: Additional file 1 — Supplementary 1 Cloud BioLinux software documentation in the form of a mini, self-contained website. Users need to download and uncompress the .zip file, and open through a web browser the "index.html" file available on the main directory. (ZIP 1823 kb). [file 1471-2105-13-42-S1.ZIP › Cloud-BioLinux-Package-Documentation/docs/maxdLoad2.html]

Bio-Linux Software Documentation Pages

Back to search form

## maxdLoad2

|  |  |
| --- | --- |
| Name | maxdLoad2 |
| Description | **maxd** is a data warehouse and visualisation environment for genomic expression data. It is being developed at the University of Manchester by the Microarray Bioinformatics Group.  **maxdLoad2** is the second generation of the database and the data loading and curation application.  **maxdLoad2** offers a simple route to MIAME compliance with MAGE-ML export options. |
| Homepage | http://bioinf.man.ac.uk/microarray/maxd/ |
| Remote Documentation | http://bioinf.man.ac.uk/microarray/maxd/maxdLoad2/index.html |
